# Takotsubo Syndrome After Alcohol Withdrawal in a Patient With Suspected Alcoholic Cardiomyopathy

**DOI:** 10.7759/cureus.57175

**Published:** 2024-03-29

**Authors:** Satoshi Kurisu, Hitoshi Fujiwara

**Affiliations:** 1 Department of Cardiology, Hiroshima-Nishi Medical Center, Otake, JPN

**Keywords:** scintigraphy, echocardiography, case report, atrial fibrillation, ventricular dysfunction

## Abstract

Takotsubo syndrome is a non-ischemic cardiomyopathy characterized by transient left ventricular (LV) apical ballooning, which typically occurs after exposure to emotional or physical stress in elderly women. An 85-year-old woman with hypertension presented with a recent onset of palpitation and exertional dyspnea. The patient had a long-standing history of alcohol consumption, and transthoracic echocardiography revealed diffuse LV hypokinesia including apical area with an ejection fraction of 30%. The patient was suspected of alcoholic cardiomyopathy and was recommended to quit alcohol consumption. Six weeks after the first admission, the patient presented to the emergency department with a three-day history of dyspnea. Based on newly developed negative T-waves and LV apical akinesia in the absence of significant coronary artery disease, the patient was diagnosed with takotsubo syndrome combined with suspected alcoholic cardiomyopathy. Clinicians should be aware that takotsubo syndrome can occur even in the presence of reduced LV ejection fraction, leading to further LV systolic dysfunction.

## Introduction

Takotsubo syndrome is a non-ischemic cardiomyopathy commonly characterized by transient left ventricular (LV) apical ballooning, which typically occurs after exposure to emotional or physical stress in elderly women [1-5]. The pathophysiological mechanism remains unclear but is likely multifactorial, consisting of multivessel coronary spasm, coronary microvascular impairment, catecholamine surge, and dysregulated corticosteroid hormone balance [1-5]. Alcohol abuse has also been implicated in its occurrence [6-9]. There is a complex relationship between alcohol drinking and stress. Alcohol has anxiety-reducing properties and can relieve stress, while at the same time acting as a stressor and activating the body’s stress response systems [10].

Herein, we report an elderly female patient with a long-standing history of alcohol consumption presenting with diffuse LV systolic dysfunction, who developed takotsubo syndrome after alcohol withdrawal.

## Case presentation

An 85-year-old woman with hypertension presented to an outpatient clinic with a recent onset of palpitation and exertional dyspnea, New York Heart Association class II. The patient had a long-standing history of alcohol consumption. The family reported that she had taken 4-6 standard drinks of Japanese rice wine (sake) per day while living apart and then a standard drink after living together in the last three months. There was no history of cardiomyopathies or sudden cardiac death in the family. The patient was admitted for cardiac evaluation.

**Figure 1:**
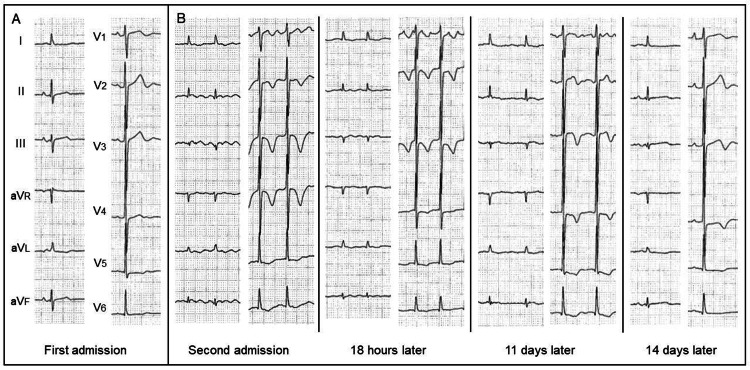
Electrocardiograms Electrocardiography (ECG) on the first admission showed mild ST-segment depression and flat T-wave in V_5-6_ leads (A). ECG on the second admission showed atrial fibrillation with newly developed negative T-waves in V_2-6_ leads. Negative T-waves were incompletely resolved on follow-up ECGs (B).

On physical examination, her pulse rate was 70 bpm, blood pressure was 110/64 mmHg, body weight was 32 kg, and body mass index was 16.8 kg/m^2^. There was no edema in the lower extremities. Laboratory data showed increased values of mean corpuscular volume (MCV), N-terminal pro-brain natriuretic peptide (NT-proBNP), and troponin-I (Table 1). Liver function, thyroid function, and thiamine level were almost normal. Electrocardiography (ECG) showed mild ST-segment depression and flat T-wave in V_5-6_ leads (Figure 1A) and atrial fibrillation with newly developed negative T-waves in V2-6 leads (Figure 1B).

**Table 1:** Laboratory data

Variable	First admission	Second admission	Reference range
White blood cell count	6.9 × 10^3^ cells/mm^3^		3.4 - 8.6 × 10^3^ cells/mm^3^
Red blood cell count	4.34 × 10^6^ cells/mm^3^		3.69 - 4.91 × 10^6^ cells/mm^3^
Hemoglobin	14.0 g/dL		11.4 - 15.1 g/dL
Hematocrit	40.7%		34.9 - 45.1%
Mean corpuscular volume	95.2 fL		85.5 - 93.5 fL
Platelet count	155 × 10^3^ cells/mm^3^		149 - 351 × 10^3^ cells/mm^3^
Total bilirubin	1.39 mg/dL	1.02 mg/dL	0.3 - 1.2 mg/dL
Aspartate aminotransferase	24 U/L	21 U/L	13 - 33 U/L
Alanine aminotransferase	15 U/L	13 U/L	6 - 27 U/L
Gamma-glutamyl transpeptidase	23 U/L	20 U/L	10 - 47 U/L
Creatine phosphokinase	66 U/L	70 U/L	45 - 163 U/L
Creatine phosphokinase-MB	7.0 U/L	5.4 U/L	0 - 5.7 U/L
Blood urea nitrogen	18.2 mg/dL	20.7 mg/dL	8 - 22 mg/dL
Creatinine	0.60 mg/dL	0.58 mg/dL	0.40 - 0.79 mg/dL
C-reactive protein	0.05 mg/dL	0.09 mg/dL	0 - 0.3 mg/dL
Troponin-I	73 pg/mL	112 pg/mL	0 - 26 pg/mL
N-terminal pro-brain natriuretic peptide	2,109 pg/mL	28,131 pg/mL	< 126 pg/mL
Triiodothyroxine	1.98 pg/mL		1.71 - 3.71 pg/mL
Thyroxine	1.54 ng/dL		0.7 - 1.48 ng/dL
Thiamine	94 ng/mL		24 - 66 ng/mL

**Figure 2:**
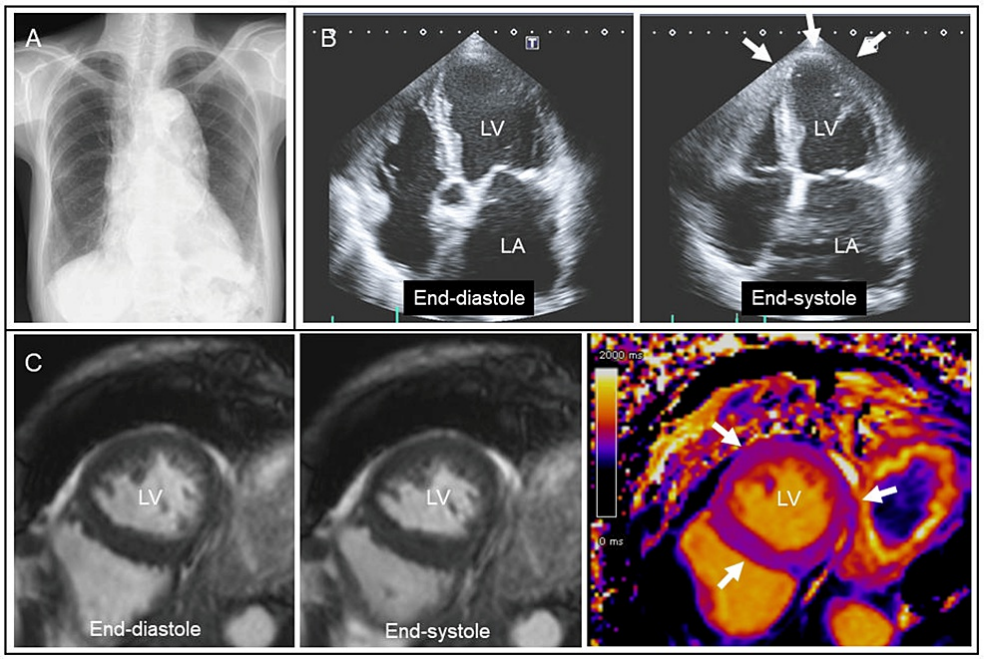
Cardiac examinations during the first hospitalization Chest radiography on the first admission showed enlarged aortic and cardiac silhouettes (A). Transthoracic echocardiography revealed diffuse LV hypokinesia including apical area with an ejection fraction of 30% (B, arrows). Cine cardiac magnetic resonance also showed diffuse LV hypokinesia with normal T1 mapping measurements (C, arrows).

Chest radiography showed enlarged aortic and cardiac silhouettes (Figure 2A). Transthoracic echocardiography revealed diffuse LV hypokinesia including apical area with an ejection fraction of 30% (Figure 2B, arrows). Considering her body surface area (1.1 m^2^), the LV end-diastolic diameter (46 mm) was increased with normal LV wall thickness (9 mm). There were no significant valvular heart diseases. Cine cardiac magnetic resonance also showed diffuse LV hypokinesia with normal T1 mapping measurements (Figure 2C, arrows). Subsequent cardiac computed tomography was negative for coronary artery disease. Holter monitoring detected paroxysmal atrial fibrillation with a rapid ventricular response. Because of a long-standing history of alcohol consumption, the patient was suspected of alcoholic cardiomyopathy and was recommended to quit alcohol consumption [11-14]. As for medications, amlodipine (2.5 mg/day) was switched to carvedilol (5 mg/day) and enalapril (2.5mg/day) with cardio-protective effects. Apixaban (5 mg/day) was added to prevent thromboembolism.

Six weeks after the first admission, the patient presented to the emergency department with a three-day history of dyspnea. NT-pro-BNP (28,131 pg/mL) and troponin-I (112 pg/mL) levels were markedly increased compared with those on first admission. ECG showed atrial fibrillation with newly developed negative T-waves in V_^2-6^_ leads (Figure 1B). Chest radiography showed mild pulmonary congestion and pleural effusion, suggesting decompensated heart failure. She was admitted again for the treatment and further cardiac evaluation.

**Figure 3:**
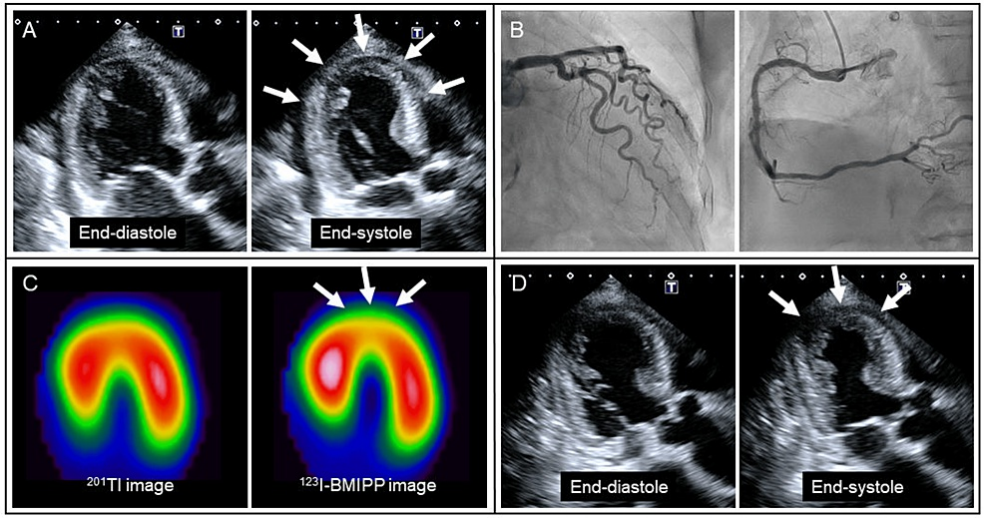
Cardiac examinations during the second hospitalization Given newly developed left ventricular (LV) apical akinesia (A, arrows), emergency coronary angiography was performed, revealing the absence of significant coronary artery disease (B). In the LV apical area, myocardial perfusion was preserved on ^201^Tl imaging, whereas fatty acid metabolism was reduced on ^123^I-BMIPP imaging (C, arrows). On hospital day 11, LV apical wall motion returned to hypokinesia (D, arrows).

Given newly developed negative T-waves (Figure 1B) and LV apical akinesia (Figure 3A, arrows), emergency coronary angiography was performed, revealing the absence of significant coronary artery disease (Figure 3B). The patient responded well to intravenous furosemide, and her symptoms disappeared. Spironolactone (25 mg/day) and dapagliflozin (5 mg/day) were further added. On hospital day 8, myocardial scintigraphy with dual isotopes of thallium-201 (^201^Tl) and iodine-123-beta-methyl-p-iodophenyl penta-decanoic acid (^123^I-BMIPP) was performed [3]. In the LV apical area, myocardial perfusion was preserved on ^201^Tl imaging, whereas fatty acid metabolism was reduced on ^123^I-BMIPP imaging (Figure 3C, arrows). There was a significant mismatch between ^201^Tl and ^123^I-BMIPP images. Taken together, the patient was diagnosed with takotsubo syndrome combined with suspected alcoholic cardiomyopathy. On hospital day 11, LV apical wall motion returned to hypokinesia (Figure 3D, arrows).

The patient’s general condition worsened mainly due to anorexia and sarcopenia. The patient passed away on hospital day 36.

## Discussion

In this report, we presented an elderly female patient with suspected alcoholic cardiomyopathy, who developed takotsubo syndrome after alcohol withdrawal.

Regular, heavy alcohol abuse can lead to alcoholic cardiomyopathy characterized by LV dilatation and systolic dysfunction with normal or reduced LV wall thickness [11-14]. In general, patients with alcoholic cardiomyopathy have a history of consuming > 80 g/day for > 5 years [11]. Although several laboratory tests such as MCV serve as markers of alcohol addiction [14], there are no specific clinical or laboratory characteristics associated with alcoholic cardiomyopathy. The diagnosis is primarily based on the association of chronic alcoholic abuse with LV systolic dysfunction that cannot be attributed to other evident heart diseases [11-14].

In the present case, the patient had no evidence of specific cardiomyopathies and coronary artery disease on cardiac magnetic resonance T1 mapping [15] and computed tomography, leading to a diagnosis of suspected alcoholic cardiomyopathy. As a unique aspect of our case, the patient was a very small, elderly woman who did not look like a heavy drinker. Women are more susceptible to developing alcoholic cardiomyopathy at smaller total lifetime doses of alcohol use [12,13]. Our case highlights the importance of medical interviews in the diagnosis of alcoholic cardiomyopathy.

The other unique aspect was that takotsubo syndrome further developed under the condition of suspected alcoholic cardiomyopathy. Takotsubo syndrome resembles anterior acute myocardial infarction in initial symptoms and ECG changes [1-5]. In the present case, computed tomography had already revealed the absence of significant coronary artery disease during the first hospitalization. Nevertheless, emergency coronary angiography was performed due to newly developed ECG and echocardiographic changes. Angiographic evaluation aided in differentiating between the two diseases promptly. The mismatch between ^201^Tl and ^123^I-BMIPP images supported the diagnosis of takotsubo syndrome [3]. In fact, several cases of takotsubo syndrome after alcohol withdrawal have been reported in both men [6,7] and women [8,9]. However, cardiac conditions before alcohol withdrawal were not described in these reports. In contrast, the present report demonstrated temporal changes in LV apical wall motion before and after alcohol withdrawal, suggesting that takotsubo syndrome can develop even under the condition of alcoholic cardiomyopathy. According to a review paper by Becker [10], chronic abuse and withdrawal experience constitute potent stressors, leading to hypothalamic-pituitary-adrenocortical axis activation and long-lasting dysregulation of the neuroendocrine stress response as well as perturbations in sympathetic nervous system activity. In the present case, the patient developed takotsubo syndrome under the use of carvedilol. Data from the International Takotsubo Registry also showed no evidence of survival benefits or reduced rate of recurrence with the use of beta-blockers [1,4]. Thus, the preventive effects of beta-blockers seem to be limited. Further studies are necessary to clarify the precise mechanism of takotsubo syndrome after alcohol withdrawal.

## Conclusions

In conclusion, we encountered an elderly female patient with suspected alcoholic cardiomyopathy, who further developed takotsubo syndrome after alcohol withdrawal. This case highlights an importance of medical interview in the diagnosis of alcoholic cardiomyopathy. Clinicians should be aware that takotsubo syndrome can occur even in the presence of reduced LV ejection fraction, leading to further LV systolic dysfunction.
